# Insights into the Mn^2+^ Binding Site in the Agmatinase-Like Protein (ALP): A Critical Enzyme for the Regulation of Agmatine Levels in Mammals

**DOI:** 10.3390/ijms21114132

**Published:** 2020-06-10

**Authors:** María-Belen Reyes, José Martínez-Oyanedel, Camila Navarrete, Erika Mardones, Ignacio Martínez, Mónica Salas, Vasthi López, María García-Robles, Estefania Tarifeño-Saldivia, Maximiliano Figueroa, David García, Elena Uribe

**Affiliations:** 1 Departamento de Bioquímica y Biología Molecular, Facultad de Ciencias Biológicas, Universidad de Concepción, Casilla 160-C, Concepción 4070386, Chile; marireyesc@udec.cl (M.-B.R.); caminavarrete@udec.cl (C.N.); ermardones@udec.cl (E.M.); ignmartinez@udec.cl (I.M.); etarisal@udec.cl (E.T.-S.); maxifigueroa@udec.cl (M.F.); dgarcia@udec.cl (D.G.); 2 Instituto de Bioquímica y Microbiología, Universidad Austral de Chile, Valdivia 5110566, Chile; monicasalas@uach.cl; 3 Departamento de Ciencias Biomédicas, Universidad Católica del Norte, Coquimbo 1781421, Chile; vjlopez@ucn.cl; 4 Departamento de Biología Celular, Facultad de Ciencias Biológicas, Universidad de Concepción, Casilla 160-C, Concepción 3349001, Chile; mgarcia@udec.cl

**Keywords:** ureohydrolase, ALP, manganese

## Abstract

Agmatine is a neurotransmitter with anticonvulsant, anti-neurotoxic and antidepressant-like effects, in addition it has hypoglycemic actions. Agmatine is converted to putrescine and urea by agmatinase (AGM) and by an agmatinase-like protein (ALP), a new type of enzyme which is present in human and rodent brain tissues. Recombinant rat brain ALP is the only mammalian protein that exhibits significant agmatinase activity in vitro and generates putrescine under in vivo conditions. ALP, despite differing in amino acid sequence from all members of the ureohydrolase family, is strictly dependent on Mn^2+^ for catalytic activity. However, the Mn^2+^ ligands have not yet been identified due to the lack of structural information coupled with the low sequence identity that ALPs display with known ureohydrolases. In this work, we generated a structural model of the Mn^2+^ binding site of the ALP and we propose new putative Mn^2+^ ligands. Then, we cloned and expressed a sequence of 210 amino acids, here called the “central-ALP”, which include the putative ligands of Mn^2+^. The results suggest that the central-ALP is catalytically active, as agmatinase, with an unaltered *K_m_* for agmatine and a decreased *k_cat_*. Similar to wild-type ALP, central-ALP is activated by Mn^2+^ with a similar affinity. Besides, a simple mutant D217A, a double mutant E288A/K290A, and a triple mutant N213A/Q215A/D217A of these putative Mn^2+^ ligands result on the loss of ALP agmatinase activity. Our results indicate that the central-ALP contains the active site for agmatine hydrolysis, as well as that the residues identified are relevant for the ALP catalysis.

## 1. Introduction

**Figure 1:**
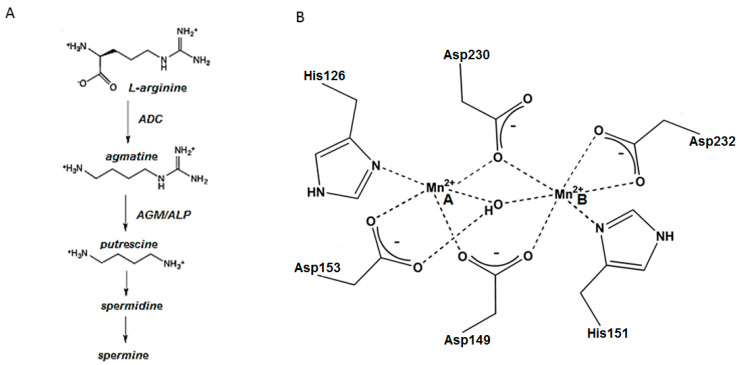
(**A**) Pathway of agmatine biosynthesis and breakdown. ADC: arginine decarboxylase; ALP: agmatinase-like protein; AGM: agmatinase. (**B**) Schematic illustration of the Mn^2+^ binding site of *E. coli* AGM. The enzyme can accommodate two closely spaced Mn^2+^ ions in their active sites, using highly conserved amino acid side chains [18].

Agmatine (1-amino-4-guanidinobutane) results from the decarboxylation of L-arginine by arginine decarboxylase (ADC) and is hydrolyzed to putrescine and urea by agmatinase (AGM) or agmatinase-like protein (ALP), shown in Figure 1A. Agmatine has been directly associated with many important cellular functions, such as the modulation of insulin release from pancreatic cells [1,2,3], renal sodium excretion [4,5], inhibition of nitric oxide synthase [6,7], neuroprotective effects [8], increased tolerance to morphine [9], modulation of ethanol anxiolysis [10], and regulation of polyamine biosynthesis [11,12]. It is considered a neurotransmitter/neuromodulator [13] because it regulates the release of catecholamines and potentiates opioid analgesia [14]. Indeed, the injection of agmatine produces anticonvulsant, antineurotoxic and antidepressant-like actions in animals [13,15]. It has also been linked to other central nervous system disorders. Specifically, preclinical studies have demonstrated the beneficial effects of agmatine administration on diseases such as depression, anxiety, hypoxic ischemia, nociception, morphine tolerance, memory, Parkinson’s disease, Alzheimer’s disease, traumatic brain injury-related disorders, and epilepsy [16,17]. The various biological processes on which agmatine is involved suggest that it might require the fine regulation of its cellular concentrations. Thus, understanding its synthesis (by ADC) and hydrolysis (by AGM or ALP) is crucial to understanding its regulation.

AGM belongs to the ureohydrolases enzyme family, which requires bivalent ions for its catalytic activity, especially Mn^2+^. On the active site, six strictly conserved amino acid residues are responsible for metal coordination; four *Asp* and two *His* residues [18,19,20], as shown in Figure 1B. While AGM from *Escherichia coli* has been extensively studied, a detailed characterization of mammalian AGM is still lacking. In this sense, our laboratory has identified a rat brain protein with significant agmatinase activity in vitro [21,22]. Interestingly, the deduced amino acid sequence of this enzyme greatly differs from all known members of the ureohydrolase family, lacking the characteristic Mn^2+^ ligands and catalytic residues [21]. Based on its agmatinase activity and the lack of sequence conservation, we referred to this enzyme as “agmatinase-like protein” (ALP).

ALP, which is present only in mammals and has been identified in rat brain tissues (astrocytes and neurons of hypothalamus and hippocampus [23]), display a *k_cat_* of 0.9 ± 0.2 s^−1^ for agmatine hydrolysis and a *K_m_* value of 3.0 ± 0.2 mM for agmatine [21,22]. Furthermore, we demonstrated its ability to generate putrescine under in vivo conditions [22,24]. From these results, and given that human-AGM does not display agmatinase activity [25,26], ALP might be the enzyme regulating agmatine concentrations in mammals and, therefore, the various important functions associated with agmatine [16,18].

A singular component of ALP protein sequence is the motif C-X16-H-X2-C-X2-C-X2-C-X21-C-X2-C (X denotes any amino acid) in the C-terminus (residues 459–510). This motif is characteristic of the so-called LIM-domain, commonly found in mammalian proteins and involved in protein–protein interactions [27,28]. We have shown that a deletion mutant of ALP, lacking the LIM-domain, is catalytically more active than the wild-type ALP; the truncated variant (∆LIM-ALP) exhibits a 10-fold higher *k_cat_* and a three-fold lower *K_m_* value for agmatine [22,26]. In this study, ∆LIM-ALP is used as a positive control in the characterization of ALP variants.

Regarding the metal ion requirements in ALP agmatinase activity, we have determined that ALP requires Mn^2+^ ions for its activity, and the presence of EDTA produces a total inactivation, which is reverted by the addition of metal ions [21,29]. In this respect, ALP behaves similar to all Mn^2+^-dependent members of the ureohydrolase family, such as the *E. coli* AGM [30,31] and arginases (ARG) [32,33,34]. In their fully active state, these enzymes contain a binuclear Mn^2+^ center, which, according to our results, is also present in ALP [29,35,36]. Due to the lack of structural information and the low degree of sequence identity between ALP and all known ureohydrolases, the active site in ALP is completely unknown. As we mentioned earlier, in ureohydrolases, aspartate and histidine amino acids are ligands for the metallic cofactor. However, mutations of the five *His* residues in ALP did not generate significant changes except for the mutant H206A, which produced a 10-fold decreased affinity for Mn^2+^ binding [29]. These results indicate that, in contrast with AGM and ARG, histidine residues are not critical for the catalytic activity of ALP.

In the present study, we identified the active site region of ALP and proposed the residues required for Mn^2+^ binding.

## 2. Results and Discussion

### 2.1. Manganese Binding Site in ALP

**Figure 2:**
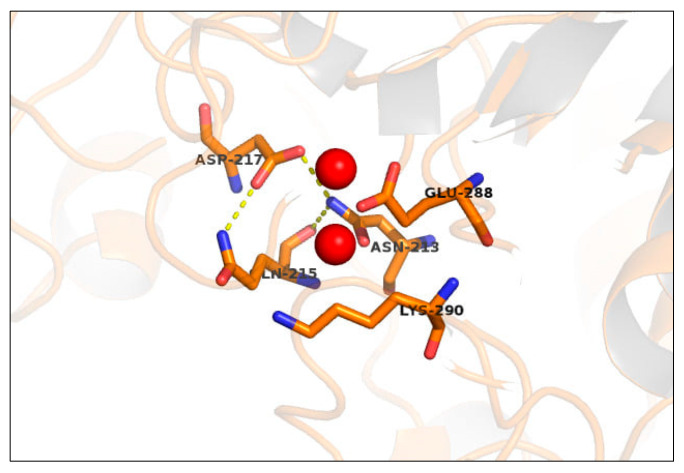
Model of the putative Mn^2+^ binding site for ∆LIM-ALP, generated through the software MODELLER 9.22. The Mn^2+^ binding site proposed including Asn213, Gln215, Asp217, Glu288, and Lys290, instead of four *Asp* and two *His* (the scheme does not include Glu 190).

Using comparative modeling we generated a structural model of ∆LIM-ALP, including the Mn^2+^ binding site, but without considering the first 30 residues and 3 longer loops (H67 to G111, E145 to S178, and E345 to P417) that represent the principal sequence singularity of this protein. The model passed the stereochemistry and energy conformation assessment, as described in Section 4.2. The model presents the general folding of this protein family, presenting only differences in the length of some of the secondary structure elements. As shown in Figure 2, the amino acids present in the putative Mn^2+^ binding site of ALP contain numerous variations regarding the conserved amino acid in the ureohydrolase family. In general, in metalloproteins, metal ions are coordinated by donor groups as nitrogen, oxygen, or sulfur centers belonging to the amino acid residues of the protein. There are three “major binders” of Mn^2+^ ions: oxygen atoms from carboxyl groups of aspartic and glutamic acids side chains, and imidazole nitrogen atoms from histidine side chain. Minor binders are: oxygen atoms from hydroxyl groups of serine and threonine side chains; amide nitrogen and oxygen atoms from asparagine and glutamine side chains; sulfur atoms from thiol group of cysteine and thioether group of methionine and oxygen atoms from peptide bonds of all the amino acids, including even hydrophobic ones [37].

We suggest that in ALP the Mn^2+^ interactions involve a new type of ligation between E190, N213, Q215, D217, E288, and K290 and the Mn^2+^ ions; similar residue types interact with Mn^2+^ ions in others proteins [38]. While aspartate is often found stabilizing binuclear metal centers, residues such as glutamate and asparagine can also play this role [35]. For example, it has been reported that Asn81 stabilizes the binuclear Mn^2+^ center of metallophosphoesterase from a marine bacteria [39], and Asn233 plays a similar role in the binuclear Zn^2+^ center of the betalactamase of *Bacillus cereus* [40]. Furthermore, glutamic residues (Glu235 and Glu204) have been described as stabilizing the binuclear Co^2+^ center of a methionine aminopeptidase from *E. coli* [35] and the binuclear Mn^2+^ center (Glu 56-57-58) of a pyrophosphohydrolase from *E. coli* [41]. On the other hand, Gln and Lys residues have not been described with such a role, however, Gln displays similar physicochemical properties to Asn, and Lys has been linked to the second coordination sphere interactions of metal ions [42].

Based on our structural comparative model and the literature supporting the stabilizing role of the residues identified, we suggest that in ALP, the Mn^2+^ interactions are performed by residues which are not the classic *Asp* and *His* found in the ureohydrolases enzyme family [18].

### 2.2. Expression and Characterization of Central-ALP

**Figure 3:**
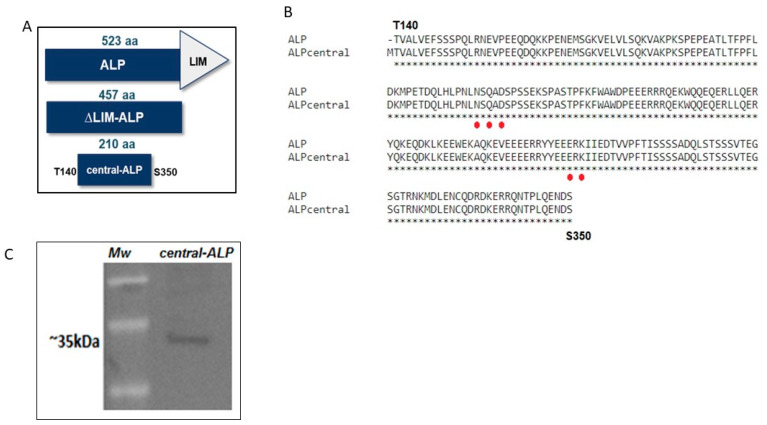
(**A**). Comparative scheme of ALP, ∆LIM-ALP, and central-ALP. The regions shown in blue are identical between the different proteins. (**B**). Alignment of central-ALP construction and ALP, the proposed metal ligands are indicated in red. (**C**). Western blot analysis of central-ALP obtained from N^2+^-NTA chromatography. A band of approximately 35 kDa corresponding to central-ALP was observed. An anti-ALP antibody dilution 1:2000 was used.

**Figure 4:**
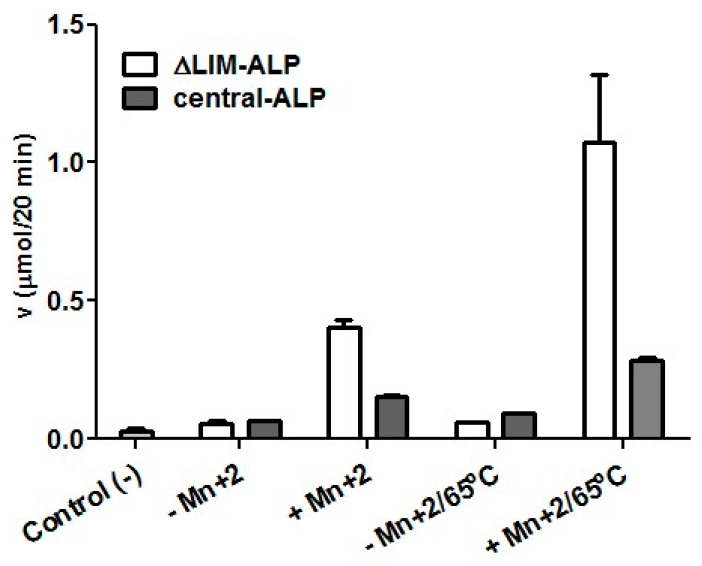
Effect of Mn^2+^ on the agmatinase activity of central-ALP and ∆LIM-ALP. Empty bars indicate ∆LIM-ALP activity and black bars indicate central-ALP activity; -Mn^2+^ indicates the measurement of activity in the absence of added manganese; +Mn^2+^ indicates the measurement of activity with 5 mM MnCl_2_. In addition, activity was measured with previous treatment of incubation at 65 °C for 5 min, in the presence and absence of 5 mM Mn^2+^, then the tubes were cooled and the agmatinase activity was determined at 37 °C. Negative control (−), measured with only 80 mM agmatine in 50 mM glycine–NaOH (pH 9.0) and 5 mM MnCl_2_.

To study the putative Mn^2+^ binding site and define the region containing the active site of ALP, we focused our analysis on 210 amino acid regions of ALP (from T140–S350) flanking the putative Mn^2+^ binding site, as shown in Figure 3 (complete sequence of ALP is in Supplementary Materials). This region was selected to minimize the disruption of the secondary structure predicted by our model, and it was called the central-ALP variant due to its location in the WT-ALP, as shown in Figure 3A,B. The sequence of central-ALP was amplified by PCR and cloned on the H6pQE60 *E. coli* expression vector, which adds a *His*-Tag for further purification. The expressed and partially purified enzyme was confirmed by Western blot, as previously described by Mella et al. [23] and, as expected, the central-ALP variant had a molecular weight of 25 KDa, as shown in Figure 3C. Further, we performed agmatinase activity assays for central-ALP in the presence and absence of Mn^2+^. As shown in Figure 4, our results indicate that central-ALP (grey bars) did not show activity in the absence of Mn^2+^, while its activity increased two-fold when the metal ion was added to the media. We used ∆LIM-ALP as a positive control (white bars in Figure 4), this variant displays high AGM activity (10-fold higher than ALP) and its purification is simple [22]. As expected, similar results were observed with ∆LIM-ALP increasing its activity eight-fold in the presence of Mn^2+^. These results showed that central-ALP can hydrolyze agmatine and its activity is Mn^2+^ dependent.

We also studied AGM activity when the variants were heated at 65 °C in presence of Mn^2+^, cooled down to room temperature, and AGM activity was measured at 37 °C (5 mM Mn^2+^ on the media). We observed that central-ALP increased its AGM activity four-fold, while ∆LIM-ALP variant displayed 20-fold increased activity. These results agree with our previous observations where wild-type-ALP increased its activity ~4-fold [29]. The increased activity of ALP, produced for the heating at 65 °C with Mn^2+^, is a typical characteristic of ureohydrolases, such as the rat liver arginase [18,39] and *Helicobacter pylori* ARG [32]. It has been suggested that the heating of the enzyme in the presence of Mn^2+^ increases the activity of the enzyme through the stabilization of its binuclear Mn^2+^ center [32]. These results together suggest that an Mn^2+^ binuclear center is formed on the central-ALP variant and that it contains enough residues to stabilize the Mn^2+^ ions.

**Figure 5:**
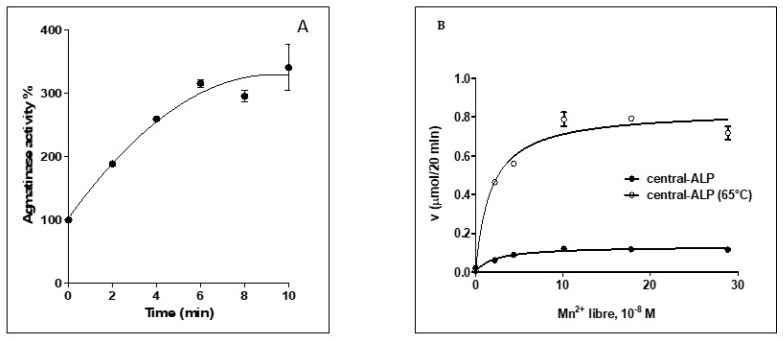
(**A**) Enzymatic activation assays of central-ALP with Mn^2+^. The incubations were carried out at 65 °C at different times, in the presence of Mn^2+^ 5 mM, then the tubes were cooled and agmatinase activity was determined at 37 °C. (**B**) Determination of the activation constant by Mn^2+^ in central-ALP. The enzyme was incubated for 15 min at 37 °C with varying concentrations of Mn^2+^ in 10 mM Tris–HCl (pH 8.5), 50 mM KCl, and 10 mM nitrilotriacetic acid as a metal ion buffer. Then, agmatine was added and incubated at 37 °C for 15 min. Then, the AGM activities were determined. The studies were performed in duplicates and repeated twice. Activation constants (*K_a_*) were estimated from the hyperbolic dependence of agmatinase activity on free-Mn^2+^ concentrations (similarly to a *K_m_* for Mn^2+^).

To study the kinetics of ALP activation by Mn^2+^, we pre-incubated central-ALP with 5 mM Mn^2+^ at 65 °C during different periods, as shown in Figure 5A. We found that central-ALP progressively increases its activity until rising to a plateau, and a similar tendency was observed for ALP [29]. Then, we measured AGM activity at different concentrations of Mn^2+^ to determine an activation constant (*K_act_* Mn^2+^), as shown in Figure 5B and Table 1. We used central-ALP in two conditions, previously heated at 65 °C in the presence of Mn^2+^ and without pre-heating. In both cases, the constant was similar to ΔLIM-ALP, as shown in Table 1. This *K_act_* has been directly associated with the dissociation constant (*K_d_*) of the enzyme–Mn^2+^ complex [18]. Therefore, we suggest that the affinity for Mn^2+^ is maintained in central-ALP and the ligands required to coordinate the metal ions are present on this variant.

**Figure 6:**
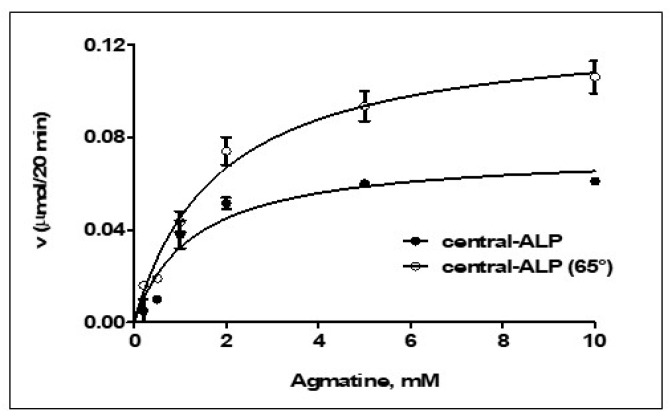
Michaelis–Menten plot for central-ALP. The saturation curve for central-ALP previously heated to 65 ° C for 5 min is observed in white circles and in black circles without heating. The activities were performed at 37 °C and at pH 9.0 with MnCl_2_ 5 mM and different agmatine concentrations.

The kinetic characterization of central-ALP showed a Michaelis–Menten saturation curve, shown in Figure 6, in both conditions, previously heated with Mn^2+^ at 65 °C and without heating. The *K_m_* of central-ALP was 1.8 mM (for heated assay) and 1.2 mM (without heating), which is similar to the *K_m_* of ΔLIM-ALP (1.2 mM). As seen in Table 1, the *k_cat_* of central-ALP decreased by half when compared to the wild-type-ALP. Therefore, central-ALP displays similar *K_m_* but differs in catalytic efficiency. These results suggest that central-ALP might bind to the substrate as it does to ALP-WT, however, its catalysis might require residues outside the central region to be similarly efficient.

### 2.3. Site-Directed Mutagenesis of Putative Mn^2+^ Ligands

**Figure 7:**
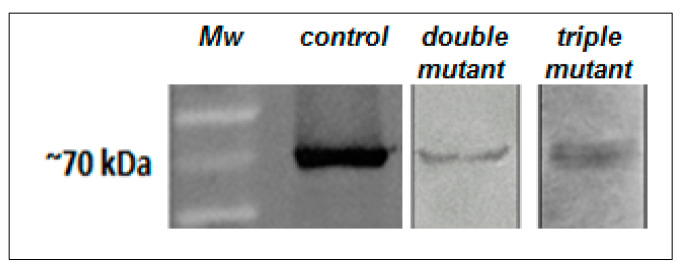
Western blot identification of mutated ∆LIM-ALP variants. Using Western blot using an anti-ALP antibody (dilution 1:2000), a band of approximately 70 kD was detected corresponding to the wild-type (control), the double mutant, and triple mutant of ∆LIM-ALP.

Finally, we performed a functional analysis of the putative Mn^2+^ ligands in ALP, identified in the present study. To do so, we generated a simple mutant D217A, a double mutant E288A/K290A, and a triple mutant N213A/Q215A/D217A of ∆LIM-ALP. These variants were expressed and identified in chromatography fractions by means of Western blot using a specific anti-ALP antibody, shown in Figure 7 [23,24]. As shown in Table 1, we did not observe agmatinase activity in these ALP mutants. The results indicate that these residues are required for the ALP activity. These findings are in agreement with observations performed for ureohydrolases, such as the rat and human arginase, and the *E. coli* agmatinase. For those enzymes, the mutation of one residue coordinating Mn^2+^ causes partial or total loss of the enzymatic activity [43,44]. For example, on *E. coli* agmatinase, the mutation of the ligand H126N reduced agmatinase activity by 50%, while the mutation H151N produced a total loss of activity [44].

## 3. Conclusions

In the present work, we conclude that the active site of the ALP enzyme resides in the central-region (from T140–S350). This region contains the ligands necessary for Mn^2+^ binding and catalysis. The fact that ALP is a ureohydrolase, is Mn^2+^-dependent, and displays such sequence divergence suggests that we are studying a new type of ureohydrolase with a new type of Mn^2+^ ligand. Our model proposes new residues for the Mn^2+^ binding site and the mutants’ results indicate the importance in the agmatinase activity in ALP. Finally, the crystal structure of ALP is required to validate our model and support our findings concerning Mn^2+^ binding.

Considering that ALP has emerged as a central enzyme in regulating crucial neurological processes, a detailed understanding of its interaction with Mn^2+^ and how its activity is controlled will be essential in defining this enzyme as a promising drug target to treat human afflictions [35,45].

## 4. Materials and Methods

### 4.1. Materials

Agmatine, glycine, Tris, SDS, and all other reagents were of the highest quality commercially available (most from Sigma Aldrich Chemical Co. Louis, MO, USA). Restriction enzymes, as well as enzymes and reagents for PCR, were obtained from Invitrogen Co. (Carlsbad, CA, USA). The synthetic nucleotide primers were obtained from the Fermelo Biotec Co. (Santiago, Chile).

### 4.2. Molecular Modeling

For the putative Mn^2+^ binding site, a molecular model for ∆LIM-ALP was generated through the software MODELLER 9.22 [46]. Because the searching for templates through Blastp server (https://blast.ncbi.nlm.nih.gov/) and threading servers, such as Genthreader (http://bioinf.cs.ucl.ac.uk/psipred/) and Phyre2 (http://www.sbg.bio.ic.ac.uk/~phyre2), did not give any suitable results, we selected members of the ureohydrolase family available in the PDB server (https://www.rcsb.org/): *Deinococcus radiodurans* (PDB id: 1WOH, 22% identity, 33% similarity, 1.75 Ǻ resolution), *Clostridium difficile* (PDB id: 3LHL, 22% identity, 32% similarity, 2.3 Ǻ resolution), *Burkholderia thailandensis* (PDB id: 4DZ4, 22% identity, 31% similarity, 1.7 Ǻ resolution) all agmatinases and a guanidine butyrase from *Pseudomonas aeruginosa* (PDB id: 3NIO, 22% identity, 33% similarity, 2.0 Ǻ resolution), and a proclavaminate amidino hydrolase from *Streptomyces clavuligerus* (PDB id: 1GQ6, 22% identity, 33% similarity, 1,75 Ǻ resolution), several of them including Mn^2+^. The alignment of the templates was done by Clustal using BLOSUM45 matrix, and was improved by structural alignment to shift the gaps to zones free of secondary structure elements. Finally 30 models were construct and assessed by DOPE. The final model was evaluated with Procheck (https://servicesn.mbi.ucla.edu/PROCHECK/) for stereochemistry and Prosa Server (https://prosa.services.came.sbg.ac.at/) for energetic assessment. The residues forming the site were proposed through structural alignment with the templates that included Mn^2+^ in the structure.

### 4.3. Enzyme Preparations

The sequence of central-ALP was amplified using the PCR technique (with Kod, Merck, high fidelity DNA-polymerase) from the plasmid H6pQE60-29.2, containing the ALP cDNA as the template. The desired sequence was confirmed by automated DNA sequence analysis. The amplified fragment of 630 bp (210 aa), was directionally cloned into the histidine-tagged pQE60 bacterial expression vector, and the histidine-tagged proteins were expressed in *E. coli* strain JM109, following induction with 0.5 mM isopropyl-β-D-thiogalactopyranoside. The central-ALP was partially purified by means of DEAE-cellulose anion exchange chromatography (calibrated with Tris-HCl 10 mM, pH 7.5), eluted with KCl 250 mM and an NTA–Ni^2+^ affinity chromatography. The purity of all preparations was ~70%. The single mutant D217A, the double mutant E288A/K290A, and the triple mutant, N213A/Q215A/K290A of the putative metal-ligand site of ALP were obtained by using the QuikChange^®^ Site-Directed Mutagenesis Kit (Stratagene) with the plasmid H6pQE60-29.2, containing the ∆LIM-ALP cDNA as the template. The presence of the desired mutation and the absence of unwanted changes were confirmed by automated DNA sequence analysis.

### 4.4. ALP Activity Determination

Routinely, the ALP activities were determined by measuring the formation of urea (product) using 80 mM agmatine in 50 mM glycine–NaOH (pH 9.0) and 5 mM MnCl_2_. All the assays were initiated by adding the enzyme to the substrate, buffer, and MnCl_2_ solution, which were previously equilibrated at 37 °C. The urea was determined by the formation of a colored complex with α-isonitrosopropiophenone [33], measuring the absorbance at 540 nm. Initial velocity studies were performed in duplicates and repeated three times. Kinetic parameters were obtained by fitting the experimental data to the appropriate Michaellis–Menten equation (*vi* = *V*max*S*/*Km* + *S*) by using nonlinear regression with Graph Pad Prism version 7.0 for Windows (Graph Pad Software Inc., San Diego, CA, USA). Protein concentration was determined using the standard Bio-Rad protein assay (Bio-Rad, Hercules, CA, USA) with bovine serum albumin as standard.

### 4.5. Enzyme–Metal Interactions Analysis

For reactivation assays with Mn^2+^, the enzyme was incubated for 15 min at 37 °C with varying concentrations of Mn^2+^ in 10 mM Tris–HCl (pH 8.5), 50 mM KCl, and 10 mM nitrilotriacetic acid as a metal ion buffer. Then, agmatine was added and incubated at 30 °C for 15 min and the AGM activities were determined. The studies were performed in duplicates and repeated twice. Activation constants (*K_a_*) were estimated from the hyperbolic dependence of agmatinase activity on free-Mn^2+^ concentrations, using nonlinear regression analysis in Graph Pad Prism 5.0 (similar to a *K_m_* for Mn^2+^). Free Mn^2+^ concentrations were calculated using a dissociation constant of 3.98 × 10^−8^ M and a pKa value of 9.8 for nitrilotriacetic acid (NTA) using the software MaxChelator WINMAXC 2.4 (http://www.stanford.edu/~cpatton/maxc.html) [36].

### 4.6. Statistical Analysis

The results were evaluated with GraphPad Prism v7.0 using an analysis of variance (ANOVA), multiple comparison tests, or an unpaired two-tailed t-test.

## Figures and Tables

**Table 1:** Kinetic parameters of agmatine hydrolysis of ALP variants.

	*K_m_* (mM)	*k_cat_* (s^−^¹)	*K_act_ Mn^2+^* (M)
ALP-wt	3.0 ± 0.20	0.9 ± 0.2	1.88 × 10^−^^8^
∆LIM-ALP	1.2 ± 0.04	10 ± 1	3.6 × 10^−^^8^
∆LIM-ALP/D217A	No activity	No activity	No activity
∆LIM-ALP/E287A/K289A	No activity	No activity	No activity
∆LIM-ALP/N213A/Q215A/D217A	No activity	No activity	No activity
Central-ALP	1.2 ± 0.37	0.4 ± 0.5	1.7 × 10^−^^8^
Central-ALP (65 °C)	1.2 ± 0.37	0.8 ± 0.4	2.2 × 10^−^^8^

## Supplementary Materials

Supplementary materials can be found at https://www.mdpi.com/1422-0067/21/11/4132/s1.
