# Supplementary material for: Insights into the Mn2+ Binding Site in the Agmatinase-Like Protein (ALP): A Critical Enzyme for the Regulation of Agmatine Levels in Mammals
Source: Int J Mol Sci. 2020 Jun 10;21(11):4132. doi: 10.3390/ijms21114132 (PMC7313459; doi:10.3390/ijms21114132)
Supplement: Supplementary file 1 [file ijms-21-04132-s001.pdf]

Supplementary Material:

ALP sequence:

>ALP
MLTPRPYSQPKNSQEVLKTFKVDGKVSMNGETARGDVEGKEKEDPTAVAPGPSLTKSQMFEATVHGSPVQVK
QGSNSIEINIKKPNSPPQELTAASEETESNGRDDENGEESSGARDVELDSAEPQHFTTTVTRCSPTVALVEFSSSP
QLRNEVPEEQDQKKPENEMSGKVELVLSQKVAKPKSPEPEATLTFPFLDKMPETDQLHLPNLNSQADSPSSEKS
PASTPFKFWAWDPEEERRRQEKWQQEQERLLQERYQKEQDKLKEEWEKAQKEVEEEERRYYEEERKIIEDTVVP
FTISSSSADQLSTSSSVTEGSGTRNKMDLENCQDRDEERRQNTPLQENGSDSSLKARESGLPEERSSLTQSPSANS
ENSVSKGISQDQQPETEAEASHCGTNPQSAQDPPWNQQISNPPTSKSEDVKPKTLALEKSINHQIESPGERRKSI
SGKKLCSSCGLALGKGAAMIIETLNLYFHIQCFRCGICKGQLGDAVSGTDVRIRNGLLNCTDCYMRSRSAGQPTTL
